# Supplementary material for: Cell-mediated cytotoxicity within CSF and brain parenchyma in spinal muscular atrophy unaltered by nusinersen treatment
Source: Nat Commun. 2024 May 15;15:4120. doi: 10.1038/s41467-024-48195-3 (PMC11096380; doi:10.1038/s41467-024-48195-3)
Supplement: Supplementary file 1 — Supplementary Information [file 41467_2024_48195_MOESM1_ESM.pdf]

Supplementary Methods

Flow Cytometry of Peripheral blood mononuclear cells (PBMCs)

PBMCs thawed from previously cryopreserved samples were thawed in a 37°C water bath for 8 min. The cell suspension was transferred to a 50 ml conical tube and 9 ml pre-warmed RPMI-medium (RPMI (Sigma Aldrich), 10% FCS Gold Plus (BioSell), 1% Glutamax (Gibco), 1% Na-Pyruvate (Invitrogen)) was added prior to centrifugation at 300 g for 10 min. Supernatant was discarded and the cell pellet was resuspended in RPMI-medium. PBMC were counted and viability was assessed using a Countess II automated cell counter (Invitrogen). Subsequently, PBMC were subjected to functional immune phenotyping by flow cytometry. For this purpose, PBMC were directly stained with fluorochrome-conjugated antibodies (Table S11) raised against lineage defining epitopes (CD3, CD4, CD8, CD45RO, CD27, CD56, CD16) for 20 min at room temperature followed by intra-cellular/-nuclear staining (Granzyme A/B/K/M, Perforin) with fluorochrome-conjugated antibodies (Supplementary Data 12) in Perm/Fix buffer (BD Biosciences) for 30 min at 4°C. Samples were acquired on a Cytoflex flow cytometer (Beckman Coulter) under daily quality control by CytoFlex Daily QC Fluorospheres (Beckman Coulter). Data resulting from flow cytometric investigations was analyzed by Kaluza 2.1 (Beckman Coulter) and FlowJo 10.8.1 (BD Biosciences).

Single nuclei pathology sequencing (snPATHO-seq)

Two 25-µm thick formalin-fixed paraffin-embedded (FFPE) sections were used for nuclei isolation according to the Miltenyi FFPE Tissue Dissociation Kit protocol (Miltenyi Biotec). A total of 150,400 nuclei in a single nuclei suspension were hybridized overnight at 42°C with Human WTA Probes (10X Genomics) and loaded onto the Chromium Controller using the Fixed RNA Profiling Reagent Kits (10X Genomics). Next generation sequencing libraries with dual-indexes were sequenced on a local Illumina Nextseq 2000 with a 28-10-10-90 read setup. Processing of the sequencing data was performed with the CellRanger pipeline v7.1.0 (10X Genomics) according to the manufacturer's instructions. Briefly, raw BCL files were de-multiplexed using the CellRanger *mkfastq* pipeline. Subsequent read alignment to the Human_Transcriptome_Probe_Set_v1.0.1 and transcript counting were done using the CellRanger *multi* pipeline with standard parameters. Details regarding sequencing depth and nuclei recovery are provided in Suppl. Tab. 1. To integrate the Visium data with the snPATHO-seq result, data was first normalized with SCTransform and then we applied the FindTransferAnchors and TransferData functions in Seurat. Based on the prediction scores, we identified cell types and their spatial distribution by the FindSpatiallyVariableFeatures function.

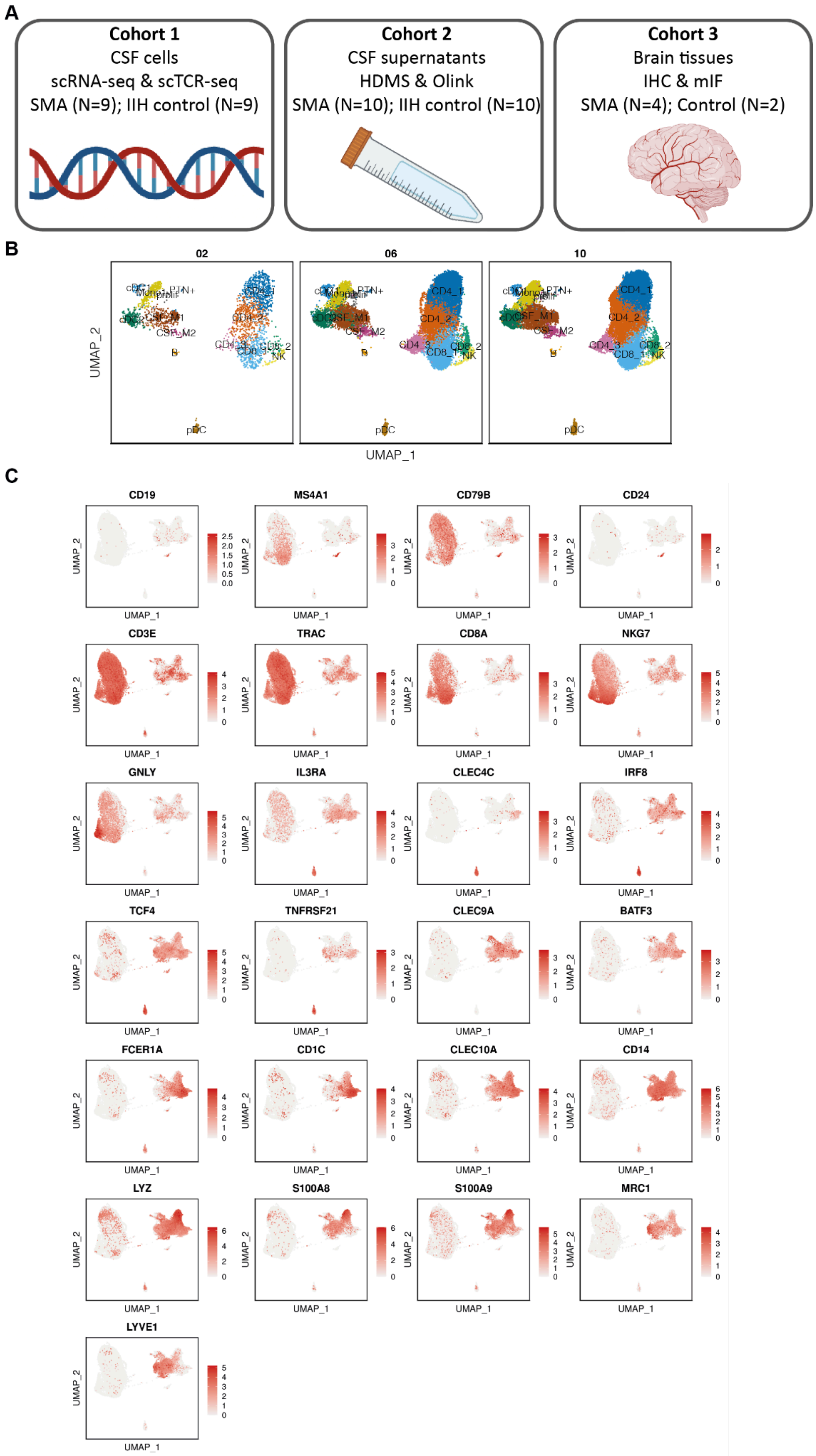
A
Cohort 1
CSF cells
scRNA-seq & scTCR-seq
SMA (N=9); IIH control (N=9)
Cohort 2
CSF supernatants
HDMS & Olink
SMA (N=10); IIH control (N=10)
Cohort 3
Brain tissues
IHC & mIF
SMA (N=4); Control (N=2)
B
02
06
10
UMAP_2
UMAP_1
CD4_1
CD4_2
CD4_3
CD8_1
CD8_2
NK
CSF_M1
CSF_M2
B
pDC
C
CD19
MS4A1
CD79B
CD24
CD3E
TRAC
CD8A
NKG7
GNLY
IL3RA
CLEC4C
IRF8
TCF4
TNFRSF21
CLEC9A
BATF3
FCER1A
CD1C
CLEC10A
CD14
LYZ
S100A8
S100A9
MRC1
LYVE1
UMAP_1
UMAP_2

Supplementary Figure 1. Details on the study cohorts and the cell cluster features based on scRNA-seq data. (A) Tree cohorts were used to assess immune responses in SMA. The diagram was created with BioRender.com and Adobe Illustrator. **(B)** UMAP plots showing 15 color-coded cell clusters of CSF cells from treated SMA patients split by month-post-treatment. 02: SMA_ 2mo; 06: SMA_6mo; 10: SMA_10mo. **(C)** Feature genes of cell clusters are shown. Color encodes average gene expression.

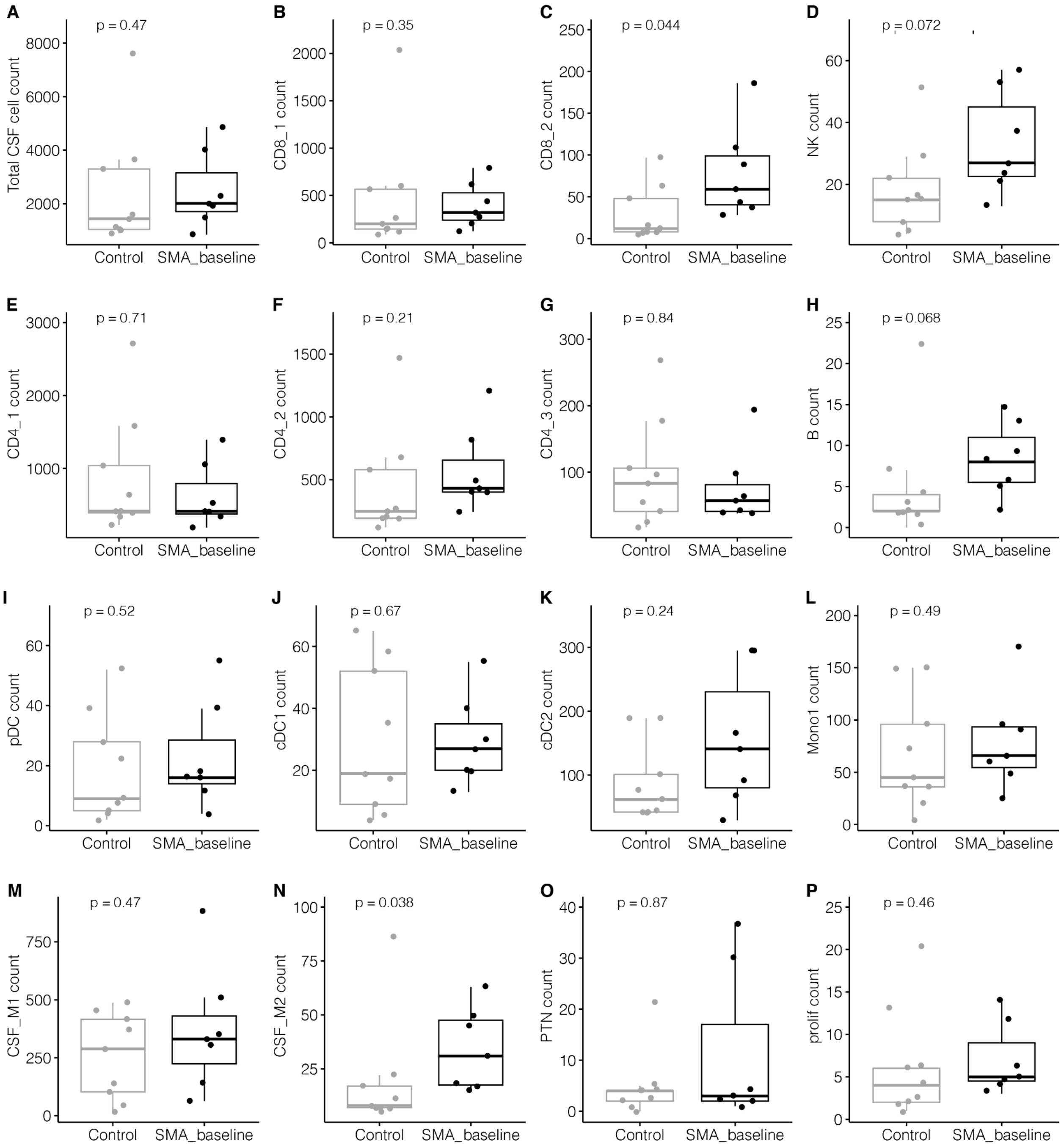

Supplementary Figure 2. Comparing cerebrospinal fluid absolute cell counts in Control and SMA_baselin. (A) Total CSF cell counts in 5 mL of CSF in Control (N=5) vs in SMA_baseline (N=7). **(B-P)** Cell counts of each CSF cell cluster in 5 mL of CSF in Control (N=5) vs in SMA_baseline (N=7). Data are depicted as median, and the lower quatile and upper quartile. Whisker include 1.5 times the interquartile range. Two-tailed Wilcoxon rank-sum test is used to calculate the p-values.

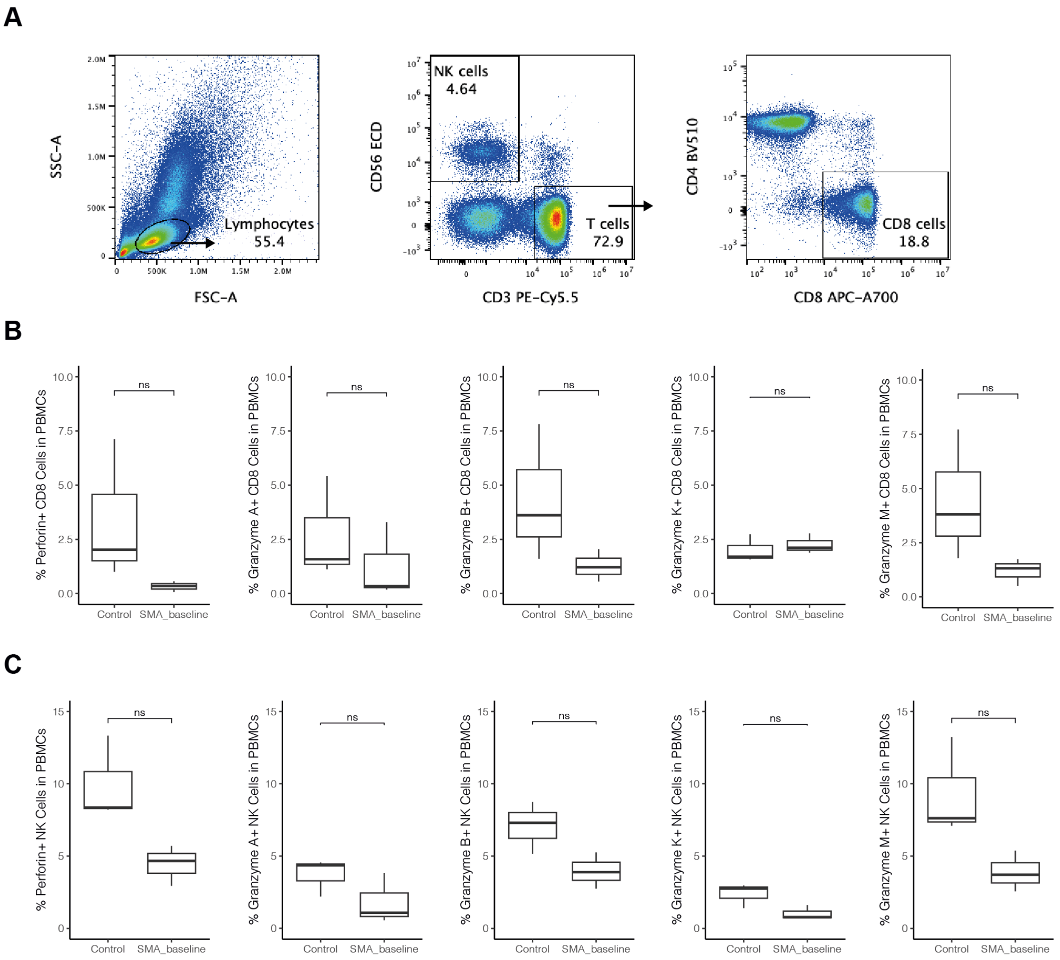

Supplementary Figure 3. Flow cytometry analysis of cryo-preserved peripheral blood mononuclear cells (PBMCs). (A) Gating strategy of CD8 cells and NK cells. **(B)** Percentage of perforin+ / granzyme A+ / granzyme B+ / granzyme K+ / granzyme M+ CD8 cells in the PBMCs. **(C)** Percentage of perforin+ / granzyme A+ / granzyme B+ / granzyme K+ / granzyme M+ NK cells in the PBMCs. Two-tailed Wilcoxon rank-sum test was used to calculate statistical significance between Control (N = 3) and SMA_baseline (N = 3); ns: adjusted p-value > 0.05.

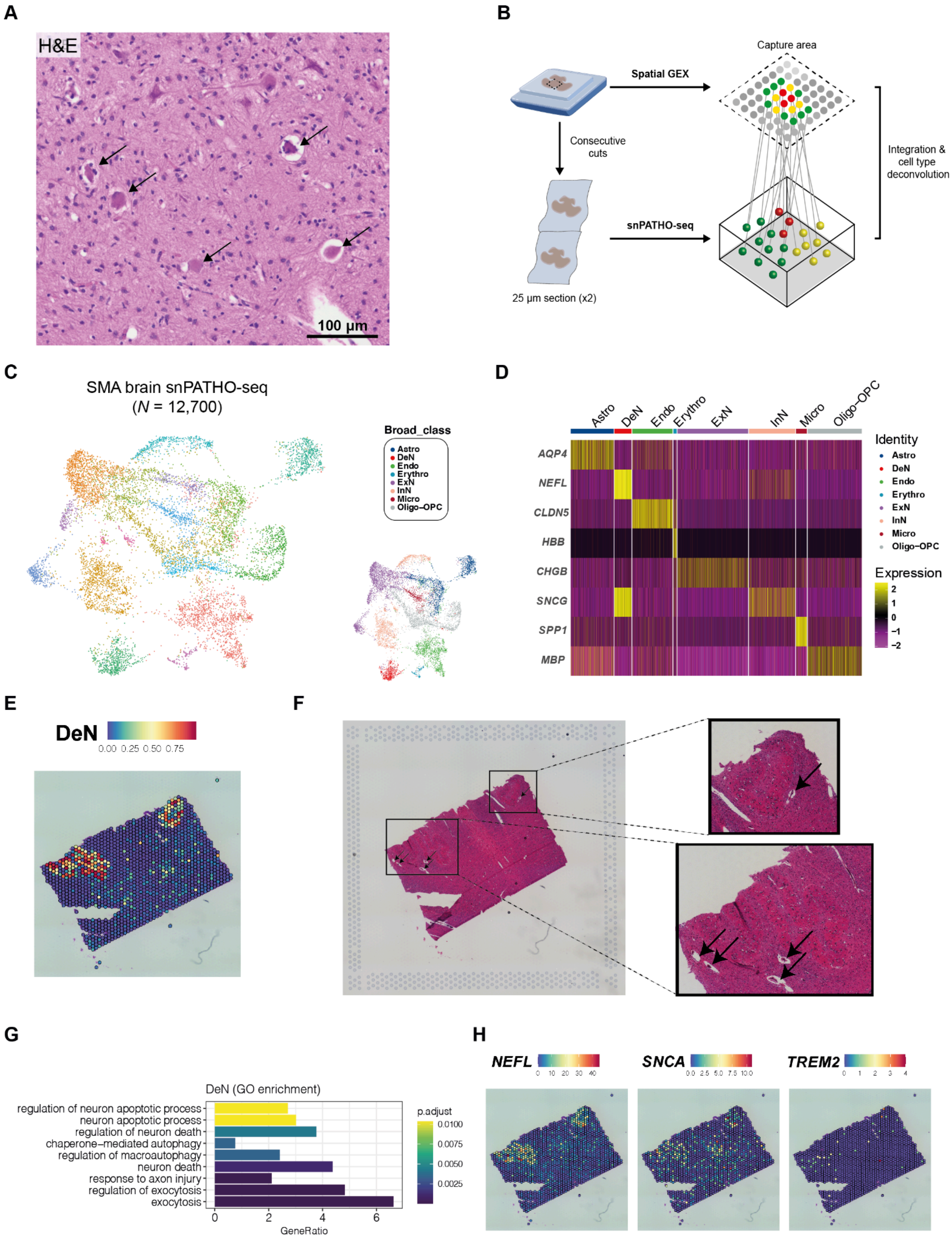
A
H&E
100 µm
B
Capture area
Spatial GEX
Consecutive cuts
Integration & cell type deconvolution
snPATHO-seq
25 µm section (x2)
C
SMA brain snPATHO-seq
(N = 12,700)
Broad_class
Astro
DeN
Endo
Erythro
ExN
InN
Micro
Oligo–OPC
D
Astro
DeN
Endo
Erythro
ExN
InN
Micro
Oligo–OPC
AQP4
NEFL
CLDN5
HBB
CHGB
SNCG
SPP1
MBP
Identity
Astro
DeN
Endo
Erythro
ExN
InN
Micro
Oligo–OPC
Expression
2
1
0
−1
−2
E
DeN
0.00 0.25 0.50 0.75
F
G
DeN (GO enrichment)
regulation of neuron apoptotic process
neuron apoptotic process
regulation of neuron death
chaperone−mediated autophagy
regulation of macroautophagy
neuron death
response to axon injury
regulation of exocytosis
exocytosis
0
2
4
6
GeneRatio
p.adjust
0.0100
0.0075
0.0050
0.0025
H
NEFL
0 10 20 30 40
SNCA
0.0 2.5 5.0 7.5 10.0
TREM2
0 1 2 3 4

Supplementary Figure 4. Multimodal spatial transcriptomics of SMA brain identifies cytotoxic neuronal damage. (A) H&E image of a post-mortem brainstem of an infant with SMA type I. Arrows highlight chromolytic neurons. **(B)** Scheme illustrating the experimental workflow. Adjacent tissue sections covering the capture area of Visium (dashed lines) in (A) were used to generate single nuclei pathology sequencing (snPATHO-Seq) and spatial gene expression (GEX) data. The diagram was created and assembled in BioRender and Adobe Illustrator. **(C)** UMAP representation of broad cell type classes (right) and 18 cell subtypes of the snPATHO-Seq dataset. **(D)** Heatmap depicting marker gene expression. Colors on the top correspond to the broad cell type classes in (C). **(E)** Characterization of spatial GEX data using cell type deconvolution. DeN: Degenerative Neuron. **(F)** H&E image derived from the same tissue section utilized for spatial transcriptomics; black arrows indicate blood vessels in close proximity to DeN. **(G)** GO terms for biological processes enriched in the DEG in the DeN cluster. **(H)** Spatial GEX of neuron degeneration related genes: *NEFL, SNCA, TREM2*.

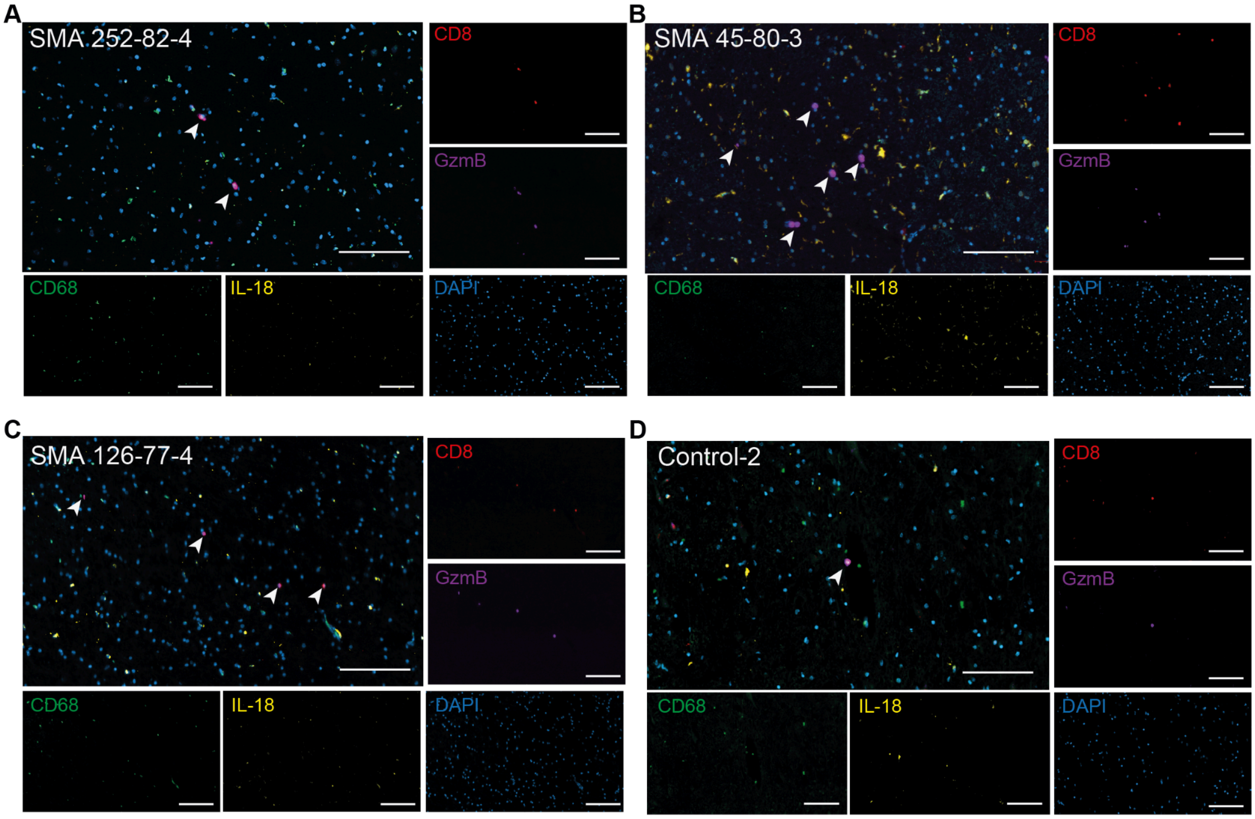

Supplementary Figure 5. Multiplexed immunofluorescence. Staining of CD8 (red), Granzyme B (GzmB; violet), IL-18 (yellow), and CD68 (green) in brain tissue sections of SMA **(A-C)** and Control **(D).** White arrowheads indicate $GzmB^{+}CD8^{+}$ cells. Scale bar: 100 μm.

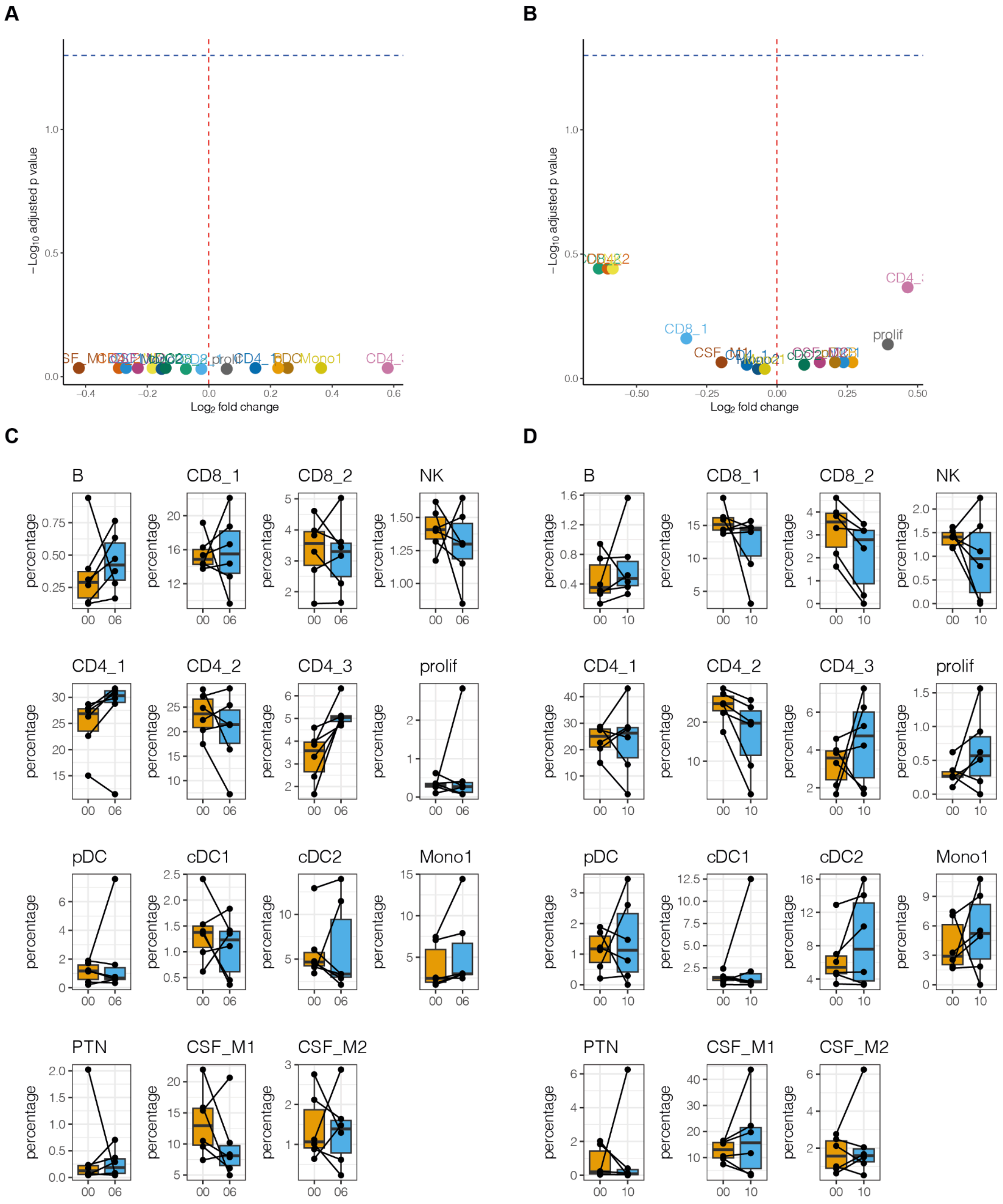

Supplementary Figure 6. Effect of Nusinersen therapy on the CSF cells. (A-B) Volcano plot depicting changes of cluster abundances in SMA_baseline versus SMA_6mo (A) and SMA_baseline versus SMA_10mo (B). Statistical significance calculated via propeller t-test, which uses a moderated t-statistic based on the robust empirical Bayes method from limma. P-values were adjusted with the Benjamini-Hoch method. Logarithmic fold change of cluster abundance is plotted against negative logarithmic p-value analyzed using propeller(logit). The horizontal lines represent the significance thresholds (blue-dashed: p = 0.05). **(C-D)** Longitudinal changes in percentage of CSF cell subtypes before and after Nusinersen treatment (N = 6); 00: SMA_baseline; 06: SMA_6mo; 10: SMA_10mo. Statistical significance calculated via the two-sideed Wilcoxon rank-sum test, as well as the Benjamini-Hochberg method for p-value adjustment. Data are depicted as median, and the lower quatile and upper quartile. Whisker include 1.5 times the interquartile range.

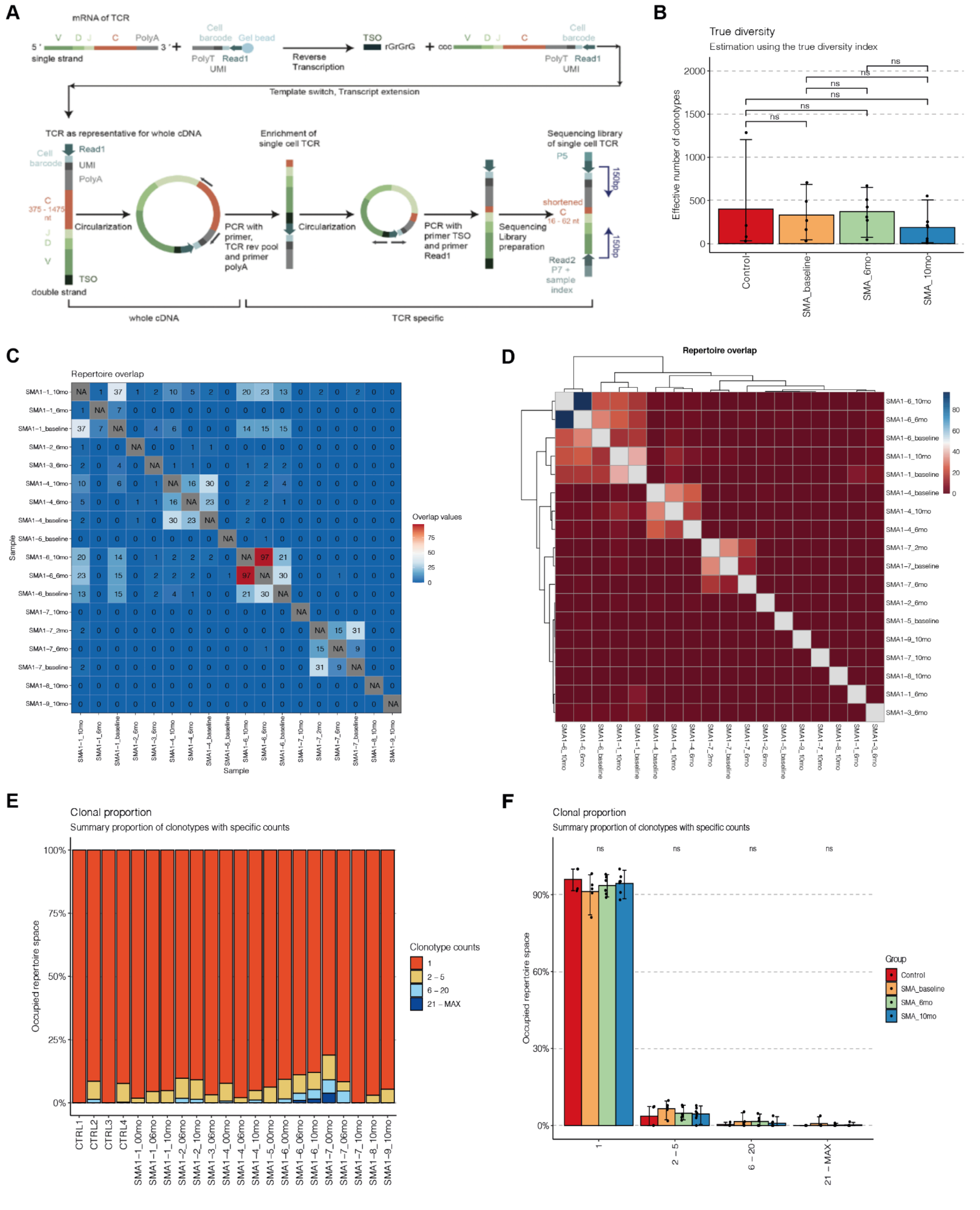

Supplementary Figure 7. Effect of Nusinersen therapy on the TCR repertoire of the total CSF T cells. (A) Scheme of sequencing antigen receptor information in 3' scRNA-seq libraries. **(B)** Diversity analysis compares the diversity of clonotypes in sample groups using the true

diversity index (Control: N=4; SMA_baseline: N=5; SMA_6mo: N=6; SMA_10mo: N=7). Individual data points are shown using overlaid dot plots. Statistical significance calculated via the Kruskal-Wallis test, as well as the Holm-Bonferroni correction method for p-value adjustments. Mean + SD are shown. **(C)** Overlap analysis of CDR3 sequences reveals the number of shared clonotypes between samples. **(D)** Hierarchical clustering analysis examines repertoire similarity. The heatmap represents the highest (dark blue) to the lowest (dark red) overlap values. **(E)** Clonality analysis compares the proportion of the individual sample occupied by the clonotypes of given sizes. **(F)** Comparison of the differences in clonotypes proportion between the sample groups (Control: N=4; SMA_baseline: N=5; SMA_6mo: N=6; SMA_10mo: N=7). Individual data points are shown using overlaid dot plots. Statistical significance calculated via the Kruskal-Wallis test, as well as the Holm-Bonferroni correction method for p-value adjustments. Mean + SD are shown.

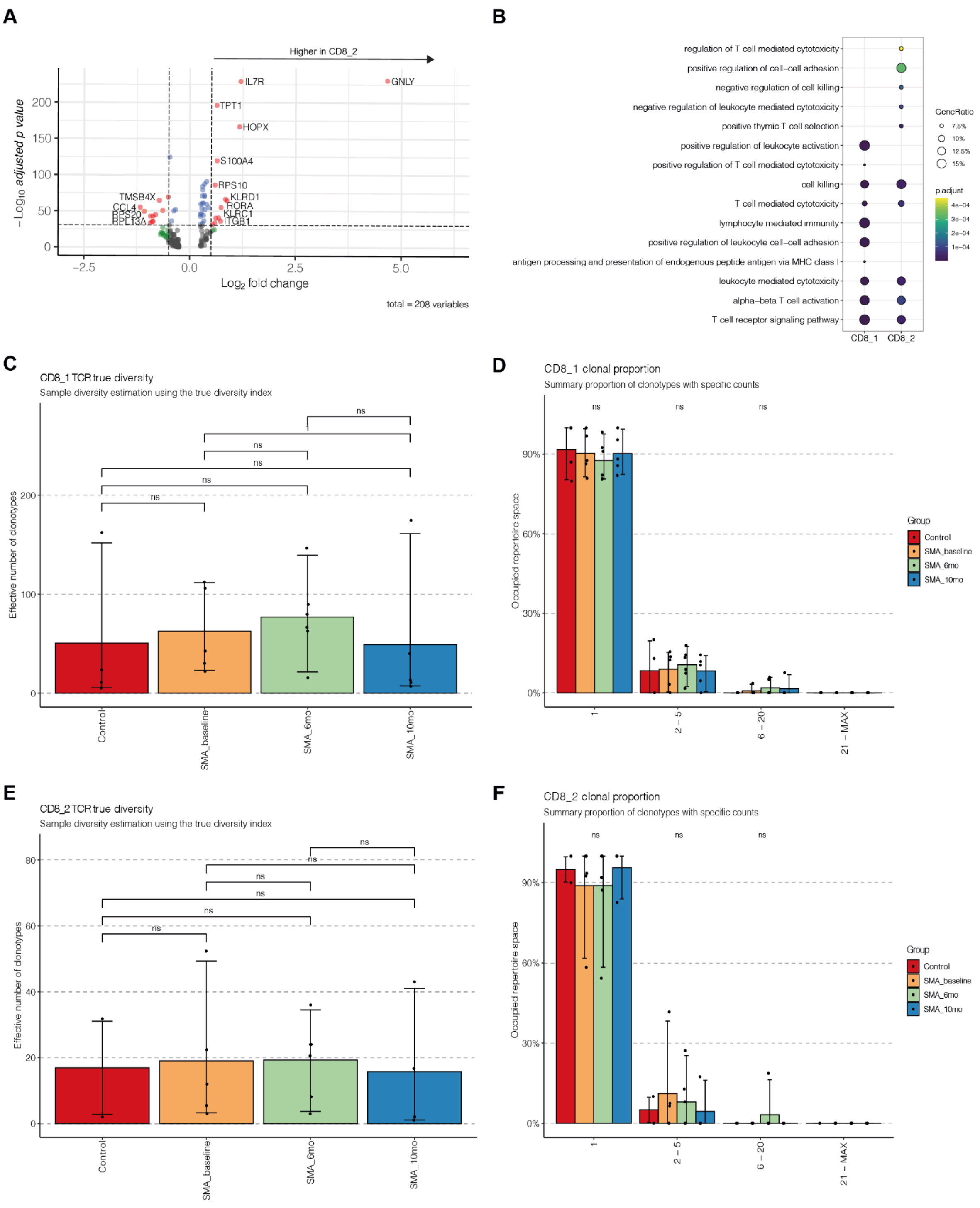

Supplementary Figure 8. CD8_1 and CD8_2 clusters. (A) Volcano plot depicting differentially expressed genes (DEG) in CD8_1 versus CD8_2 clusters. Statistical significance calculated via the two-sided Wilcoxon rank sum test, as well as the Benjamini-Hochberg method for p-value adjustment. **(B)** GO terms for biological processes enriched in the DEG in CD8_1 and CD8_2 clusters. **(C, E)** TCR diversity analysis applying the true diversity index in CD8_1 (Control: N=4; SMA_baseline: N=5; SMA_6mo: N=6; SMA_10mo: N=5) and CD8_2 (Control: N=2; SMA_baseline: N=5; SMA_6mo: N=6; SMA_10mo: N=4) clusters. Individual data points are

shown using overlaid dot plots. Statistical significance calculated via the Kruskal-Wallis test, as well as the Holm-Bonferroni correction method for p-value adjustments. **(D, F)** TCR clonality analysis in CD8_1 (Control: N=4; SMA_baseline: N=5; SMA_6mo: N=6; SMA_10mo: N=5) and CD8_2 (Control: N=2; SMA_baseline: N=5; SMA_6mo: N=6; SMA_10mo: N=4) clusters. Individual data points are shown using overlaid dot plots. Statistical significance calculated via the Kruskal-Wallis test, as well as the Holm-Bonferroni correction method for p-value adjustments. Mean + SD are shown.

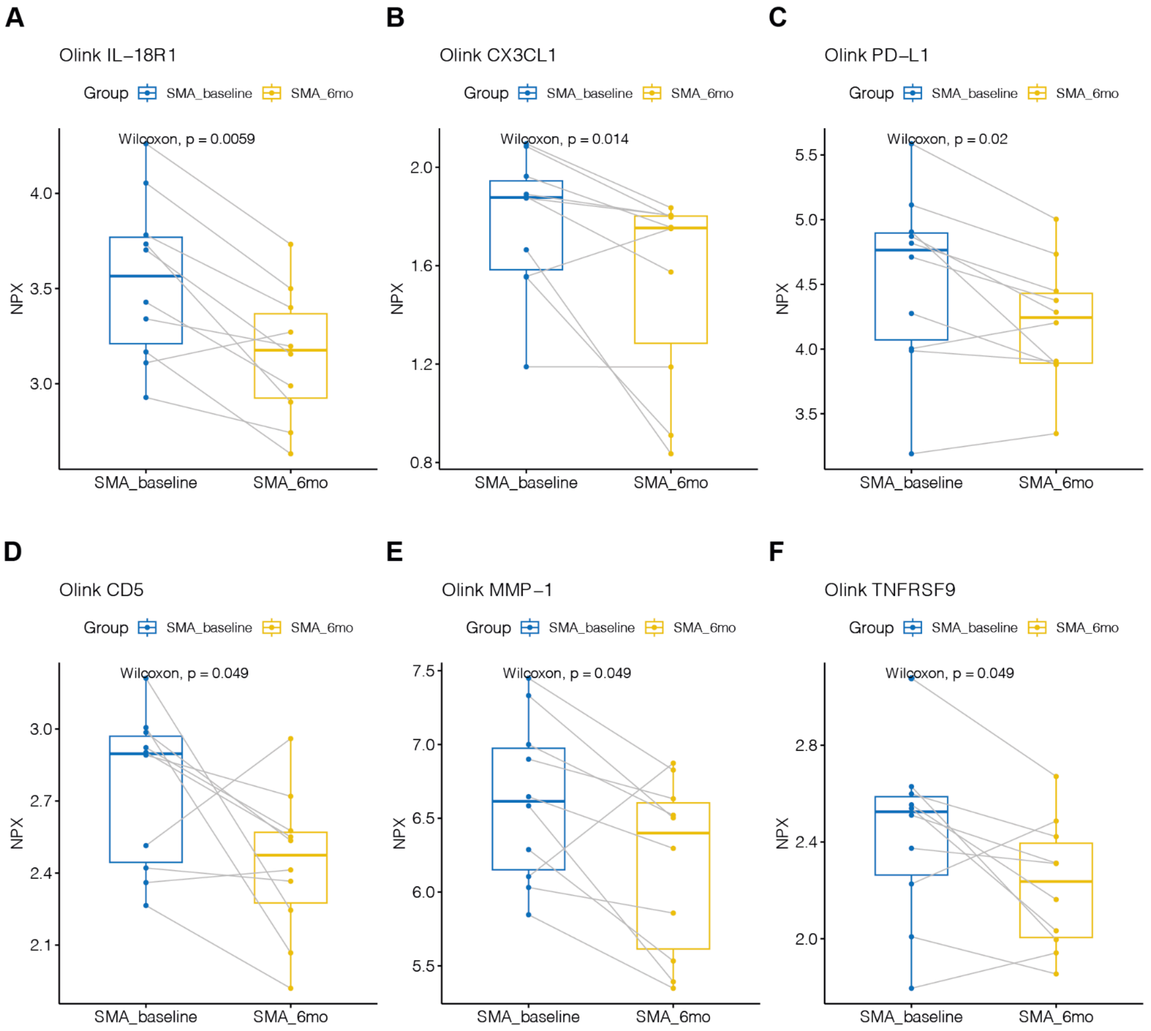

G

**Protein**	***P* value, Wilcoxon signed-ranks test**	**Adjusted *p* value, Benjamini-Hochberg**	**Adjusted *p* value, Holm's and Hochberg's**
IL-18R1	0.0059	0.2695	0.5332
CX3CL1	0.0137	0.3512	1.0000
PD-L1	0.0195	0.3512	1.0000
CD5	0.0488	0.3512	1.0000
MMP-1	0.0488	0.3512	1.0000
TNFRSF9	0.0488	0.3512	1.0000

Supplementary Figure 9. Effect of Nusinersen therapy on the CSF proteins. (A-F) Olink Normalized Protein eXpression (NPX) of IL-18R1 (A), CX3CL1 (B), PD-L1 (C), CD5 (D), MMP-1 (E), TNFRSF9 (F) in the CSF. **(G)** Statistical significance calculated via the two-sided Wilcoxon rank sum test, as well as the Benjamini-Hochberg method and the Holm's and Hochberg's family-wise error rate method for p-value adjustments.
