# Supplementary material for: Cell-mediated cytotoxicity within CSF and brain parenchyma in spinal muscular atrophy unaltered by nusinersen treatment
Source: Nat Commun. 2024 May 15;15:4120. doi: 10.1038/s41467-024-48195-3 (PMC11096380; doi:10.1038/s41467-024-48195-3)
Supplement: Supplementary file 3 — Description of Additional Supplementary Files [file 41467_2024_48195_MOESM3_ESM.pdf]

Description of Additional Supplementary Files

File Name: Supplementary Data 1
Description: Technical information on single-cell RNA sequencing. (1) scRNA-seq: Technical information on single-cell RNA sequencing. (2) snPATHO-seq & Visium: Technical information on single nuclei pathology sequencing (snPATHO-seq) and Visium. (3) scTCR-seq: Technical information on single-cell T cell receptor sequencing.

File Name: Supplementary Data 2
Description: Top marker genes of all clusters in scRNA-seq data. Average log2FC threshold was set to 0.25 and the minimal percentage of expressing genes in either of the two populations to 0.1. Statistical significance calculated via the two-sided Wilcoxon rank sum test, as well as the Bonferroni method for p-value adjustment. All genes with an adjusted p-value above 0.05 were removed. Only genes with a positive average log2 fold change were retained. avg_log2FC: natural log2 fold change of the average expression between the corresponding cluster and all remaining clusters; pct.1: percentage of cells with the gene detected in the corresponding cluster pct.2: percentage of cells with the gene detected in all remaining clusters; p_val_adj: adjusted p-value based on Bonferroni method.

File Name: Supplementary Data 3
Description: . Differentially expressed genes (DEG) of CD8_2 and NK clusters in SMA CSF. (1) DEG of CD8_2 in SMA_baseline vs Control. (2) DEG of NK in SMA_baseline vs Control. Statistical significance calculated via the two-sided Wilcoxon rank sum test, as well as the Benjamini-Hochberg method for p-value adjustment. All genes with an adjusted p-value above 0.05 were removed. A positive average log2FC value shows a higher expression in the CD8_2 or NK cluster. All mitochondrial and ribosomal genes were removed.

File Name: Supplementary Data 4
Description: Inferred cell-cell communications by CellChat. The average expressions of all predicted ligands and receptors in each cell subpopulation in (1) Control, (2) SMA_baseline, (3) SMA_6mo and (4) SMA_10mo. The significant interactions between two cell groups are identified using a permutation test by randomly permuting the group labels of cells, and then recalculating the communication probability between cell groups through a pair of ligand and receptor by CellChat analysis. The interactions with p-value <0.05 are considered significant.

File Name: Supplementary Data 5
Description: Top marker genes of all clusters in snPATHO-seq data. Statistical significance calculated via the two-sided Wilcoxon rank sum test, as well as the Bonferroni method for p-value adjustment. Average log2FC threshold was set to 0.25 and minimal percentage of expressing genes in either of the two populations to 0.25. All genes with an adjusted p-value

above 0.05 were removed. Only genes with a positive average log2 fold change were retained. avg_log2FC: natural log2 fold change of the average expression between the corresponding cluster and all remaining clusters; pct.1: percentage of cells with the gene detected in the corresponding cluster pct.2: percentage of cells with the gene detected in all remaining clusters; p_val_adj: adjusted p-value based on Bonferroni method.

File Name: Supplementary Data 6
Description: Effect of Nusinersen therapy - statistical significance by Wilcoxon signed-rank test. Wilcoxon signed-rank test was used to calculate statistical significance between paired samples: (1) SMA_baselinel vs SMA_6mo; (2) SMA_baselinel vs SMA_10mo.

File Name: Supplementary Data 7
Description: Differentially abundant (DA) proteins measured by LC-MS. (1) DA of CSF proteins in Control vs SMA_baseline. (2) DA of CSF proteins in Control vs SMA_6mo. (3) DA of CSF proteins in SMA_baseline vs SMA_6mo. Comparative data analysis on 7 Control, 8 SMA_baseline and 5 SMA_6mo were analyzed by Progenesis for Proteomics (Nonlinear Diagnostics/Waters Corp.) using the human reviewed Uniprot database (UP000005640, Jan. 2021). Differences between SMA_baseline and SMA_6mo were analyzed using paired t-test; differences between Control and SMA_baseline/SMA_6mo were analyzed using two-way ANOVA test.

File Name: Supplementary Data 8
Description: Quantification of CSF proteins by Olink Target 96 Inflammation kit. (1) NXP: quantification of 92 predefined proteins measured with the Olink Target 96 Inflammation kit. Values are shown in the Normalized Protein eXpression (NPX) unit, (2) Two-way Wilcoxon signed-ranks test: comparison of 92 inflammatory proteins before vs after 6 months of nusinersen treatment using non-parametric statistics (Wilcoxon signed-ranks test) with Benjamini-Hochberg-adjusted p-values (p_adjust), (3) Inflammatory markers: proteins and ranges of measurements included in the Olink statistical analyses.

File Name: Supplementary Data 9
Description: TCR clonal frequency.

File Name: Supplementary Data 10
Description: CNS tissues for histological analysis

File Name: Supplementary Data 11
Description: Sequential multiplexed immunofluorescence staining protocol.

File Name: Supplementary Data 12
Description: List of antibodies used in flow cytometry.
