# Supplementary material for: Cell-mediated cytotoxicity within CSF and brain parenchyma in spinal muscular atrophy unaltered by nusinersen treatment
Source: Nat Commun. 2024 May 15;15:4120. doi: 10.1038/s41467-024-48195-3 (PMC11096380; doi:10.1038/s41467-024-48195-3)
Supplement: Supplementary file 16 — Reporting Summary [file 41467_2024_48195_MOESM16_ESM.pdf]

nature portfolio

Corresponding author(s): Gerd Meyer zu Hörste, Tim Hagenacker

Last updated by author(s): Mar 27, 2024

Reporting Summary

Nature Portfolio wishes to improve the reproducibility of the work that we publish. This form provides structure for consistency and transparency in reporting. For further information on Nature Portfolio policies, see our Editorial Policies and the Editorial Policy Checklist.

Statistics

For all statistical analyses, confirm that the following items are present in the figure legend, table legend, main text, or Methods section.

n/a	Confirmed	
☐	☒	The exact sample size (*n*) for each experimental group/condition, given as a discrete number and unit of measurement
☐	☒	A statement on whether measurements were taken from distinct samples or whether the same sample was measured repeatedly
☐	☒	The statistical test(s) used AND whether they are one- or two-sided<br>*Only common tests should be described solely by name; describe more complex techniques in the Methods section.*
☐	☒	A description of all covariates tested
☐	☒	A description of any assumptions or corrections, such as tests of normality and adjustment for multiple comparisons
☐	☒	A full description of the statistical parameters including central tendency (e.g. means) or other basic estimates (e.g. regression coefficient) AND variation (e.g. standard deviation) or associated estimates of uncertainty (e.g. confidence intervals)
☐	☒	For null hypothesis testing, the test statistic (e.g. *F*, *t*, *r*) with confidence intervals, effect sizes, degrees of freedom and *P* value noted<br>*Give P values as exact values whenever suitable.*
☒	☐	For Bayesian analysis, information on the choice of priors and Markov chain Monte Carlo settings
☒	☐	For hierarchical and complex designs, identification of the appropriate level for tests and full reporting of outcomes
☒	☐	Estimates of effect sizes (e.g. Cohen's *d*, Pearson's *r*), indicating how they were calculated

Our web collection on statistics for biologists contains articles on many of the points above.

Software and code

Policy information about availability of computer code

Data collection	CytExpert v2.6 for flow cytometry data acquisition; Zeiss ZEN 3.3 Imaging Software for multiplex immunfluorescence
Data analysis	Illumina basecall (.bcl) data were converted and demultiplexed to FASTQ files using the bcl2fastq v2.20 software. RNA-Sequencing results were analyzed using the 10x Genomics Cell Ranger 6.1.0 pipeline and Seurat version 4.3 and 5.0. Bioinformatic and statistical data analysis was performed in the R environment, version 3.5.4 and 4.2.3. Some statistical analyses were performed using Prism software (v 5.0.3, GraphPad). Image data were analyzed using HALO software version 3.2.1851.371 with HighPlex FL module 4.0.4.0. Progenesis QI and Olink® Insights Stat Analysis APP were used for proteomics. Flow cytometric investigations was analyzed by Kaluza 2.1 (Beckman Coulter) and FlowJo 10.8.1 (BD Biosciences). Figures were created and assembled in BioRender and Adobe Illustrator 2020 v24.

For manuscripts utilizing custom algorithms or software that are central to the research but not yet described in published literature, software must be made available to editors and reviewers. We strongly encourage code deposition in a community repository (e.g. GitHub). See the Nature Portfolio guidelines for submitting code & software for further information.

Data

Policy information about availability of data

All manuscripts must include a data availability statement. This statement should provide the following information, where applicable:

- Accession codes, unique identifiers, or web links for publicly available datasets
- A description of any restrictions on data availability
- For clinical datasets or third party data, please ensure that the statement adheres to our policy

All single-cell sequencing data, along with associated metadata detailing clusters and treatments, can be accessed publicly through the Gene Expression Omnibus (GEO) repository, identified by reference number GSE232391.

Research involving human participants, their data, or biological material

Policy information about studies with human participants or human data. See also policy information about sex, gender (identity/presentation), and sexual orientation and race, ethnicity and racism.

Reporting on sex and gender	We provide sex and gender information of all human donors in Table 1.
Reporting on race, ethnicity, or other socially relevant groupings	All donors were of caucasian ethnicity.
Population characteristics	Population characteristics are outlined in Table 1.
Recruitment	All patients for CSF collection were recruited and all samples were collected at the Department of Neurology, University Hospital Essen and at the Department of Neurology with Institute of Translational Neurology, University Hospital Münster. All patients gave written informed consent to sample collection and data analysis. Recruitment was performed by asking patients receiving first Nusinersen treatment (= SMA baseline) whether they wanted to participate in the study and donate excess CSF for scientific purposes. By design, this was limited to patients undergoing first intrathecal treatment which could introduce bias by enriching for patients aiming to be treated. The recruitment was also limited to adult patients because the study was performed in adult Neurology departments. Pediatric patients were thus not included in the study which constitutes bias.<br><br>Pathology samples were collected at the Division of Neurochemistry and Neuropathology, Medical University of Vienna, Austria and at the Institute of Neuropathology, University Hospital Münster, Germany. This was limited to autopsy material from severely affected patients which does not reflect the less severe disease courses.
Ethics oversight	The study was approved by the local ethics committee in Essen (Ethics Committee of the University Duisburg-Essen; reference number 18-8285-BO), in Münster (Ethics Committee of the Board of Physicians of the Region Westfalen-Lippe and of the Westfälische Wilhelms-University Münster; reference 2015-522-f-S), and in Vienna (Ethics Committee of the Medical University of Vienna; reference EK 1454/18; 1636/2019).

Note that full information on the approval of the study protocol must also be provided in the manuscript.

Field-specific reporting

Please select the one below that is the best fit for your research. If you are not sure, read the appropriate sections before making your selection.

[x] Life sciences [ ] Behavioural & social sciences [ ] Ecological, evolutionary & environmental sciences

For a reference copy of the document with all sections, see nature.com/documents/nr-reporting-summary-flat.pdf

Life sciences study design

All studies must disclose on these points even when the disclosure is negative.

Sample size	For patient-derived samples, we were limited by tissue availability, and sample size was considered adequate based on comparisons with data reported from other studies in the field that used similar sample sizes.
Data exclusions	Three CSF samples of SMA patients were excluded from scRNA-Seq because blood contaminations were visually detectable.
Replication	- Single cell RNA-Seq was performed on 23 individual CSF cell samples from 7 individual SMA patients that were longitudinal (baseline: n = 7; 2mo: n = 2; 6mo: n = 6; 10mo: n = 6) and 2 additional SMA patients at 10 months post-treatment.<br>- High-definition mass spectrometry and Olink-based proteomics were performed on 10 SMA CSF supernatants and 10 IIH CSF supernatants.<br>- Multiplex immunofluorescence studies were performed on 4 SMA patients and 2 autoptic tissues.<br><br>Single cell RNA-seq samples and mass spectrometry samples were recruited and processed prospectively but not analyzed until all samples had been collected. The data analysis was then performed subsequently by combining all available data from all available patients. The analysis was thus based on human biological replicates. Inter-expeerimental replication does not apply. Each individual multiplex

	immunofluorescence staining replicated the initial observation. The number of stainings is provided in the methods section.
Randomization	In the CSF single cell RNA-seq part of the study, samples obtained from all patients who agreed to participate in the study were processed. This was an exploratory analysis not including randomization of patients or samples for two different groups of treatment or analysis. Therefore, randomization does not apply.
Blinding	The samples were processed in a non-blinded fashion but in a pseudonymized fashion. We believe that this is nearly identical to blinding and the probability for bias is very low.

Reporting for specific materials, systems and methods

We require information from authors about some types of materials, experimental systems and methods used in many studies. Here, indicate whether each material, system or method listed is relevant to your study. If you are not sure if a list item applies to your research, read the appropriate section before selecting a response.

Materials & experimental systems

n/a	Involved in the study
☐	☒ Antibodies
☒	☐ Eukaryotic cell lines
☒	☐ Palaeontology and archaeology
☒	☐ Animals and other organisms
☒	☐ Clinical data
☒	☐ Dual use research of concern
☒	☐ Plants

Methods

n/a	Involved in the study
☒	☐ ChIP-seq
☐	☒ Flow cytometry
☒	☐ MRI-based neuroimaging

Antibodies

Antibodies used

Antibody;Clone;Company;Catalogue number;Dilution
1X Opal Anti-Ms + Rb HRP Kit; Akoya Biosciences; ARH1001EA; 1:100 and 1:200
CD8; C8/144B; Agilent; GA62361-2; 1:100
Caspase-3; 5A1E; Cell Signaling Technology; 9664; 1:100
CD8; SP16; Abcam; ab101500; 1:200
CD68; KP1; Abcam; ab955; 1:200
IL-18; Polyclonal; Abcam; ab191152; 1:100
GzmB; Polyclonal; Abcamn; ab53097; 1:200
FITC-Granzyme K; GM6C3; SantaCruz; sc56125-FTIC; 1:50
PE-Granzyme B; REA226; Miltenyi; 130-116-486; 1:50
ECD-CD56; N901; Beckman Coulter; A82943; 1:50
PerCP-Cy5.5-CD3; UCHT1; Beckman Coulter; A66327; 1:50
PE-Cy7-Granzyme A; CB9; eBiosciences; 25-9177-42;1:50
eFluor660-Granzyme M; 4G2B4; eBiosciences; 50-9774-42;1:50
APC-A700-CD8; B9.11; Beckman Coulter; PN A66332; 1:50
APC-A750-CD16; 3G8; Beckman Coulter; A66330; 1:50
VioBlue-Perforin; delta G9; Miltenyi; 130-123-867; 1:50
BV510-CD4; OKT4; Biolegend; 317444; 1:50
BV605-CD45RO; UCHL1; Biolegend; 304238; 1:50
BV650-CD27; O323; Biolegend; 302828; 1:50

Validation

Antigen;Clone;Company; Reference
CD8; C8/144B; Agilent; https://www.agilent.com/cs/library/catalogs/public/00230-d58532-02-atlas-of-controls-2nd-edition-agilent.pdf
Caspase-3; 5A1E; Cell Signaling Technology; PMID: 38114471
CD8; SP16; Abcam; PMID: 30423297
CD68; KP1; Abcam; ab955; PMID: 31990684
IL-18; Polyclonal; Abcam; PMID: 30582668
GzmB; Polyclonal; Abcamn; PMID: 19895873
FITC-Granzyme K; GM6C3; SantaCruz; PMID: 16405860
PE-Granzyme B; REA226; Miltenyi; PMID: 15238416
ECD-CD56; N901; Beckman Coulter; PMID: 3884668
PerCP-Cy5.5-CD3; UCHT1; Beckman Coulter; PMID: 7796297
PE-Cy7-Granzyme A; CB9; eBiosciences; PMID: 34162888
eFluor660-Granzyme M; 4G2B4; eBiosciences; PMID: 33951417
APC-A700-CD8; B9.11; Beckman Coulter; PMID: 7821755
APC-A750-CD16; 3G8; Beckman Coulter; PMID: 2526846
VioBlue-Perforin; delta G9; Miltenyi; https://www.miltenyibiotec.com/DE-en/products/perforin-antibody-anti-human-delta-

g9.html#conjugate=vioblue:size=100-tests-in-200-ul
BV510-CD4; OKT4; Biolegend; PMID: 35151371
BV605-CD45RO; UCHL1; Biolegend; PMID: 20498045
BV650-CD27; O323; Biolegend; PMID: 34921774

Plants

Seed stocks: n/a

Novel plant genotypes: n/a

Authentication: n/a

Flow Cytometry

Plots

Confirm that:

- [x] The axis labels state the marker and fluorochrome used (e.g. CD4-FITC).
- [x] The axis scales are clearly visible. Include numbers along axes only for bottom left plot of group (a 'group' is an analysis of identical markers).
- [x] All plots are contour plots with outliers or pseudocolor plots.
- [x] A numerical value for number of cells or percentage (with statistics) is provided.

Methodology

Sample preparation: PBMCs thawed from previously cryopreserved samples were thawed in a 37°C water bath for 8 min. The cell suspension was transferred to a 50 ml conical tube and 9 ml pre-warmed RPMI-medium (RPMI (Sigma Aldrich), 10% FCS Gold Plus (BioSell), 1% Glutamax (Gibco), 1% Na-Pyruvate (Invitrogen)) was added prior to centrifugation at 300 g for 10 min. Supernatant was discarded and the cell pellet was resuspended in RPMI-medium.

Instrument: PBMC were counted and viability was assessed using a Countess II automated cell counter (Invitrogen). Samples were acquired on a Cytoflex flow cytometer (Beckman Coulter)

Software: Data resulting from flow cytometric investigations was analyzed by Kaluza 2.1 (Beckman Coulter) and FlowJo 10.8.1 (BD Biosciences).

Cell population abundance: No cell-sorting performed.

Gating strategy: Gating strategies are provided in Supplementary Figure 3.

- [x] Tick this box to confirm that a figure exemplifying the gating strategy is provided in the Supplementary Information.
